# Risk factors associated with hepatitis B exposure and the reliability of five rapid kits commonly used for screening blood donors in Ghana

**DOI:** 10.1186/1756-0500-7-873

**Published:** 2014-12-04

**Authors:** Mohamed Mutocheluh, Michael Owusu, Theophilus B Kwofie, Tahiru Akadigo, Emmanuel Appau, Patrick W Narkwa

**Affiliations:** Department of Clinical Microbiology, Kwame Nkrumah University of Science and Technology, Kumasi, Ghana; Department of Clinical Microbiology, Komfo Anokye Teaching Hospital, Kumasi, Ghana; Laboratory Department, Holy Family Hospital, Techiman, Ghana

**Keywords:** Hepatitis B virus infections, Immunochromatographic kit, Sensitivity and specificity, Blood donor samples, Ghana

## Abstract

**Background:**

Hepatitis B virus infection (HBV) is one of the most widespread, chronic viral infections in sub-Saharan Africa, and parts of South America. Therefore, efforts are being made to implement strategies aimed at reducing the incidence of hepatitis B viral infections. One route of HBV transmission is through unsafe blood transfusion, which could occur from the use of less sensitive laboratory diagnostic kits. Information on the sensitivity and specificity of these kits is however limited in many developing countries. This study was therefore performed to describe the prevalence of HBV infections and also to evaluate the performance of five rapid immunochromatographic kits commonly used in Ghana.

**Methods:**

A cross-sectional study was designed to describe the prevalence of HBsAg infection and also evaluate the performance of rapid kits used for screening hepatitis B in the northern part of Ghana.

**Results:**

A total of 164 prospective blood donors were enrolled in this study from January 2012 to December 2013. The overall true prevalence of HBsAg was 14.6 (95% CI = 9.6 – 21.0). There was no significant association between transmission related factors and HBV infection. The specificities of all five rapid kits were above 97%, however the sensitivities and Youden’s indexes were below 60%. A comparison of the reported kit sensitivities to those generated by this study showed significant difference with the study results being lower than the ones reported in the kit literature.

**Conclusion:**

Our study has shown that rapid HBsAg kits on the Ghanaian markets may not be helpful for screening blood donor samples. We therefore recommend the use of commercially available enzyme linked immunosorbent assays.

## Background

Hepatitis B virus (HBV) infection is the most widespread, chronic viral infection globally. About two billion (2 billion) people worldwide are affected and close to 350 million have active chronic HBV infection [1]. In highly endemic geographical regions such as the East and Southeast Asia, sub-Saharan Africa, and parts of South America, over 8% of the population are chronic carriers of HBV [2].

In Ghana, the prevalence rate of HBsAg has been reported to range between 8–15% in urban areas and some parts of rural Ghana; a scenario suggesting endemicity of the disease [3–5]. HBV infection therefore is an important public health concern which requires active surveillance to be conducted in other remote rural areas of Ghana.

Although surveillance of HBV infection is important for both detection and exclusion of blood donors, the utility of less sensitivity diagnostic kits could pose significant risk to patients. In Ghana and many developing countries, HBsAg screening is usually performed using rapid immunochromatographic tests (ICT). This method is preferred over the conventional assays like the enzyme linked immunosorbent assays (ELISA) because ICT kits are simple, quick, and cheaper. More so, ICT kits do not require skilled labour and are readily available on the markets. Another advantage of the ICT kits is their flexibility in terms of using both whole blood and plasma samples from subjects.

With an increasing population size and expansion of blood donor facilities to rural areas, the demand for these ICT kits has increased in Ghana and many developing countries. The scale of the influx of these ICT kits in many developing countries makes it impossible for adequate evaluation by state agencies such as the food and drugs administrations. In 2003, Allain et al., reported a 71% sensitivity of one brand of rapid dipstick among blood donors in Ghana, thus suggesting the residual risk of post-transfusion [6].

Currently many other brands of HBsAg ICT kits are on the Ghanaian market however there is insufficient data on their sensitivity and specificity. This information is useful as it will provide data to estimate the risk of transfusion related HBV infection and offer recommendations that will improve the quality of healthcare delivery in Ghana. This study was therefore undertaken to describe the prevalence of HBV and to evaluate the performance of ICT kits used in Ghana.

## Methods

### Type of study

A cross-sectional study was conducted to investigate the prevalence of hepatitis B surface antigen among blood donors and to also evaluate the performance of rapid kits used for screening blood donors.

### Study area

The cross-sectional study was carried out at the Techiman Holy Family Hospital, located in the Techiman municipality of the Brong Ahafo region in Ghana. The hospital has a current bed capacity of 211 and serves as the referral hospital for all other health facilities in the Techiman municipality and other parts of the Brong Ahafo region of Ghana. The municipality shares common boundaries with Wenchi district to the north-west, Kintampo south district to the north-east, Nkoranza district to the south-east and Offinso district in the Ashanti region to the south. The municipality has a total land area of 669.7 square kilometers with climate and vegetation that promotes the production of food. In the Brong Ahafo region, the Techiman municipality has the highest population density of 318 people per square kilometer.

### Study design and patient sampling

Two study designs were employed to describe the prevalence of HBsAg infection, identify the occurrence of HBV markers among subjects positive for HBsAg and also evaluate the performance of rapid kits used for screening hepatitis B in the northern part of Ghana.

For the cross-sectional study, a total of one hundred and sixty four (164) blood donors; made up of voluntary and replacement donors, were screened for HBsAg, between the period of January, 2012 and December, 2013. Subjects were selected if they were between 16–65 years of age, had average weight of 50 kg, haemoglobin value of 12.5 g/dl and blood pressure of less 140/90 mmHg. Subjects were excluded if they were found to be physically unfit and less than the age of 16 years. Structured questionnaires were administered to each subject selected and information on their socio-demographic status as well as possible risk factors of HBsAg exposure was retrieved from them.

For the experimental study, five brands of kits commonly used by blood donation centres to screen blood for HBsAg in the Ashanti and Brong Ahafo regional hospitals were evaluated. These brands of kits were then purchased from suppliers in the two regions and evaluated using ELISA as the gold standard. The evaluation of the test kits was done using blood samples selected at random from the cross-sectional study. Laboratory testing of the samples was carried out at the Virus Research Laboratory of the School of Medical Sciences (SMS) of the Kwame Nkrumah University of Science and Technology (KNUST) in Kumasi, Ghana.

### Sample collection and preparation

About 2 mls of ethylenediaminetetraacetic acid (EDTA) treated blood samples were collected from subjects who had reported for voluntary and replacement blood donations at the Techiman municipality. Plasma was separated from the whole blood and stored at -20°C. At the end of sampling, frozen plasma were transferred to the Virus Research Laboratory of the SMS, KNUST.

### Laboratory methods

All samples were screened for HBsAg using ELISA kits (Human Gesellschaft, Biochemica and Diagnostics, Germany).

The assay principle is based on the direct antigen ELISA technique using microwells coated with a monoclonal antibody (MAb, mouse) against HBsAg. Test samples react simultaneously with the immobilized MAb and with a polyclonal anti-HBs antibody (guinea pig) conjugated with horseradish peroxidase. If HBsAg is present in the sample, the peroxidase-containing complex is captured on the microwell surface. After incubation unbound enzyme conjugate is removed by washing. Substrate solution is added and during further incubation blue colour develops. After stopping the reaction with acidic solution the colour changes to yellow. The intensity of this colour is proportional to the amount of HBsAg in the specimen.

Testing was initiated by first bringing the kit, reagent and sera to room temperature. Testing was done according to the manufacturer’s protocol (Human Gesellschaft, Biochemica and Diagnostics, Germany). Briefly, the wells of each of the microplate were labelled for blank, controls and samples. For each batch of testing, three negative controls and two positive controls were included. One hundred microliters of wash buffer was pipetted into the blank. Fifty microliters each of negative control, positive control and test samples were pipetted into the appropriate wells. Fifty microliters of the conjugate reagent was added to each labelled well, except for the blank. It was mixed carefully and covered with Adhesive strips to prevent evaporation during incubation. The plates were incubated at 37°C for 80 minutes in an incubator. After incubation, it was washed with wash buffer for 5 times in an automatic washer. After this step, the microwell was blot dried by inverting the microplate and tapping firmly onto absorbent paper. One hundred microliter of the substrate was added to the entire microwell and mixed carefully. It was again incubated at room temperature (15-25°C) in the dark for 30 minutes. A stop solution of 100 μl was added after the time duration and the samples were read using a 450 nm filter microplate reader.

A subset of samples selected at random were further screened for HBsAg using five HBsAg ICT kits. The test was performed using guidelines provided by the manufacturer. Briefly, each strip was immersed in a well-mixed plasma specimen for 10–15 seconds. The results were read after 15–30 minutes.

Similarly, rapid HBsAg marker test was performed for all samples positive for HBsAg tests using Wonfo HBsAg panel test kit (Wondfo, China). The test was performed by pipetting 50 μl of plasma unto the test wells for 10–15 seconds. The results were read after 15–30 minutes.

### Ethical approval

The study protocol was approved by the Committee for Human Research, Publications and Ethics of Komfo Anokye Teaching Hospital (KATH) and the School of Medical Sciences, KNUST, Kumasi, Ashanti region, Ghana. The protocol was first explained to every subject that was recruited. In the case of subjects less than 18 years, the protocol was explained to their guardians or parents. Subjects who consented to the study were asked to either thumbprint or sign the participant consent form depending on whether they were literates or illiterates. Assent was obtained from those less than 18 years and written informed consent was obtained from their parents or guardians.

### Statistical analysis

Data entry was done using spreadsheet database prepared with Microsoft® Excel. Statistical analysis was done using R statistical software version 3.0.2 [7]. Socio-demographic variables such as age groups and their association with exposure to HBsAg were analyzed using the Fischer’s exact test or Chi-square test where necessary. The risk factors associated with exposure to HBsAg were determined by entering all variables that were significant at P < 0.1 from the bivariate analysis into an unconditional multiple logistic regression model. A forward and backward stepwise approach was used for selection of significant variables from the model. Results were expressed as adjusted odd ratios and 95% confidence interval (CI).

The sensitivities and specificities of the ICT kits were determined by estimating the proportion of true positive or negative test results. The reliability of the ICTs were further assessed by determining the area under the curve (AUC) using receiver operating characteristic (ROC) curve. All data manipulations were done using the EPICALC package [8] and ROC curves were plotted using the pROC package [9]. For all analysis done, a p-value of less than 0.05 was considered statistically significant.

## Results

### Characteristics of study subjects and HBsAg detections

A total of 164 prospective blood donors were enrolled in this study from January 2012 to December 2013. The median age was 28 years with a minimum of 17 years and maximum of 57 years. The number of males enrolled was higher (95.1%) than females (4.9%). The overall true prevalence of HBsAg (using the ELISA kit) was 14.6 (95% CI = 9.6 – 21.0). A comparison of ELISA tested HBsAg infections among age groups showed no significant difference (p = 0.753).

The different socio-demographic variables were also compared for subjects with HBsAg and those without exposure. There was no significant difference in the frequency of HBsAg for the subject’s occupation, marriage status, religion, type of housing and level of income. The frequency of HBsAg detection was however significant for the level of education. Table 1 describes the sociodemographic factors and their association with HBsAg detection.

**Table 1:** **Socio-demography of subjects with HBsAg positivity**

Subject information		HBsAg status			
		Positive (%)	Negative (%)	Total (%)	P-value
		24(14.6)	140(85.4)	164(100)	
Occupation of subjects	Farming	9(37.5)	30(21.4)	39(23.8)	0.527
	Teaching	0(0)	6(4.3)	6(3.7)	
	Trading	3(12.5)	26(18.6)	29(17.7)	
	Unemployed	1(4.2)	12(8.6)	13(7.9)	
	Other	11(45.8)	66(47.1)	77(47)	
Marital status of subjects	Married	15(62.5)	68(48.6)	83(50.6)	0.298
	Single	9(37.5)	72(51.4)	81(49.4)	
Religion of subjects	Christian	16(66.7)	112(80)	128(78)	0.334
	Islamic	6(25)	19(13.6)	25(15.2)	
	Traditional	0(0)	2(1.4)	2(1.2)	
	None	2(8.3)	7(5)	9(5.5)	0.334
Level of education of subjects	Illiterate	4(16.7)	11(7.9)	15(9.1)	0.028
	Junior	7(29.2)	56(40)	63(38.4)	
	Primary	9(37.5)	22(15.7)	31(18.9)	
	Secondary	4(16.7)	35(25)	39(23.8)	
	Tertiary	0(0)	16(11.4)	16(9.8)	
Type of housing of subjects	compound	14(58.3)	105(75)	119(72.6)	0.149
	Self-contained	10(41.7)	35(25)	45(27.4)	
Average income level of subjects	50	0(0)	10(7.1)	10(6.1)	0.208
	80	7(29.2)	57(40.7)	64(39.0)	
	100	12(50.0)	54(38.6)	66(40.2)	

The factors associated with exposure to HBsAg were also determined by comparing the proportion of HBsAg detections for subjects with history of drug use, intravenous drug use, tattoos, number of sexual partners and family history of liver disease. There was no significant association between HBsAg detections and the various factors (Table 2). The odds of exposure to hepatitis B was determined by entering all variables with p-value less than 0.1 into a logistic regression model. All variables were not significant except for those who attended primary school (adjusted OR = 3.05, p-value = 0.051) which was slightly at the probability borderline.

**Table 2:** **Factors associated with HBsAg exposure**

Risk of exposure to HBsAg		HBsAg			
		Positive no. (%)	Negative no. (%)	Total no. (%)	P-value
		24(14.6)	140(85.4)	164(100)	
Subjects with history of blood transfusion	No	23(95.8)	138(98.6)	161(98.2)	0.38
	Yes	1(4.2)	2(1.4)	3(1.8)	
Subjects who use IV drugs currently	No	24(100)	138(98.6)	162(98.8)	1
	Yes	0(0)	2(1.4)	2(1.2)	
Subjects with history of IV drug use	No	23(95.8)	135(96.4)	158(96.3)	1
	Yes	1(4.2)	5(3.6)	6(3.7)	
Subject with current tattoos	No	23(95.8)	139(99.3)	162(98.8)	0.275
	Yes	1(4.2)	1(0.7)	2(1.2)	
Subject with history of tattoo	No	23(95.8)	138(98.6)	161(98.2)	0.38
	Yes	1(4.2)	2(1.4)	3(1.8)	
Subjects with history of STI	No	23(95.8)	132(94.3)	155(94.5)	1
	Yes	1(4.2)	8(5.7)	9(5.5)	
Subjects with history of surgery	No	24(100)	136(97.1)	160(97.6)	1
	Yes	0(0)	4(2.9)	4(2.4)	
Number of sexual partners of subjects	More than one	6(25)	20(14.3)	26(15.9)	0.102
	None	4(16.7)	12(8.6)	16(9.8)	
	One	14(58.3)	108(77.1)	122(74.4)	
Subject with family relatives having hepatitis	No	24(100)	135(96.4)	159(97)	1
	Yes	0(0)	5(3.6)	5(3)	
Subject with family relative having liver disease	No	24(1000	137(97.9)	161(98.2)	1
	Yes	0(0)	3(2.1)	3(1.8)	

### Hepatitis B virus serological markers

HBV serological markers or biomarkers were evaluated for all study subjects who tested positive for HBsAg. The occurrence of biomarkers such as HBeAg, HBeAb, HBcAb and HBsAb were determined using rapid hepatitis B panel tests. Of the 24 subjects positive for HBsAg, 13 (54.2%) were HBcAb positive, 5 (20.8%) were HBeAb positive, 4 (16.7%) were HBeAg positive and 1 (4.2%) were HBsAb positive.

### Sensitivity and specificity of rapid kits

We evaluated the performance of the most common ICT kits used for screening blood donors in some blood bank facilities in the northern part of Ghana using the clinical samples. The sensitivity and specificity of the five ICT kits identified in the various blood bank facilities were determined using the ELISA technique as the gold standard. The rapid kit evaluation was done on the 150 samples selected at random that had already been tested using the ELISA technique. Only 150 of the 164 samples were selected for evaluation because of the limited resources available. The proportions of HBsAg that were positive for all rapid kits are as follows: Abon = 8%, Acull-Tell = 8.7%, Rapid care = 8.7%, Core TM = 8.7% and Wondfo = 9.3%. Kappa test for Wondfo was 68.7%, followed by Accul-Tell =64.7%, Care Rapid = 64.7%, Core Tm = 58.3% and then Abon = 60.6%. The Youden’s indexes for all five rapid kits were below 60%. The kit with the highest Youden’s index was Wondfo and the one with the lowest was Core Tm. All kits showed specificities and positive predictive values of more than 90%. A comparison of the performance of the rapid kits to ELISA method is shown in Table 3.

**Figure 1:**
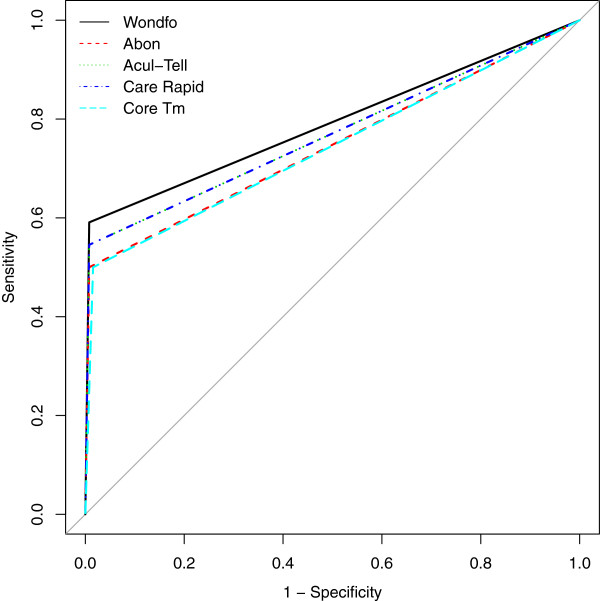
**Receiver operating characteristic curves for five immunochromatographic kits.** The x-axis shows the values for 1-specificity and the y axis, the values for sensitivity. The curves for the respective kits are as follows: Wondfo - black line, Abon - red dash line, Acul-Tell - green dotted line, Care Rapid - blue dash line and Core Tm - Cyan dash line.

To further assess the reliability of the test kits, we determined the area under the curve (AUC) for each diagnostic kit and also plotted the receiver operating characteristic (ROC) curve. Wondfo kit had the highest AUC (0.792; 95% CI = 0.686 – 0.897) followed by Acull-Tell (0.769; 95% CI = 0.662 – 0.876) and Care Rapid (0.769; 0.662 – 0.876), Abon kit (0.746; 95% CI = 0.640 – 0.853) and then Core TM (0.742; 95% CI = 0.635 – 0.850). Figure 1 shows the ROC curves for each rapid kit.

**Table 3:** **Comparative sensitivity and specificity of rapid HBsAg kit using HBsAg ELISA as gold standard**

Test kit	Sensitivity	Specificity	PPV	NPV	Youden's index	Kappa test
	(95% CI)	(95% CI)	(95% CI)	(95% CI)	(95% CI)	(%)
Wondfo	59.1(36.4 - 79.3)	99.3(95.7 - 100.0)	92.9(66.1 - 99.8)	93.4(87.8 - 96.9)	58.3(32.1 - 79.3)	68.7
Accul-Tell	54.5(32.2 - 75.6)	99.2(95.7 - 100)	92.3(64.0 - 99.8)	92.7(87.0 - 96.4)	53.8(27.9 - 75.6)	64.7
Care Rapid	54.5(32.2 - 75.6)	99.3(95.7 - 100)	92.3(64–99.8)	92.7(87.0 - 96.4)	53.8(27.9 - 75.6)	64.7
Core Tm	50(28.2 - 71.8)	98.4(94.5 - 99.8)	84.6(54.5 - 98.1)	92.0(86.1 - 95.9)	48.4(22.7 - 71.6)	58.3
Abon	50(28–71.8	99.2(95.7 - 100)	91.7(61.5 - 99.8)	92.0(86.2 - 96.0)	49.2(23.9 - 71.8)	60.6

NPV: Negative Predictive Value, PPV: Positive Predictive Value.

### Comparison of reported sensitivity and specificity of rapid kits

To further explore the performance of the rapid kits, we compared the reported sensitivities and specificities of the Accutell, Wondfo and Abon kits to results generated by this study. This comparison was done for only the three kit brands because the other kits did not have accompanying information of the kit performance in the kit inset. The three ICT kits (Accutell, Wondfo and Abon) specificities were comparable to the study results since the values fell within the confidence intervals of our specificity results. The rapid kit sensitivity values however differed significantly from the study results. Table 4 shows a comparison of the reported values of the three ICT kits compared to the results generated by this study.

**Table 4:** **Comparative sensitivity and specificity of rapid HBsAg kits**

Rapid test kit	Reported	Reported	Actual	Actual
	Sensitivity (%)	Specificity (%)	Sensitivity (95% CI)	Specificity (95% CI)
Wondfo	96.2	99.3	59.1(36.4 - 79.3)	99.3(95.7 - 100.0)
Accutell	98.89	98.87	54.5(32.2 - 75.6)	99.2(95.7 - 100)
Abon Kit	99%	97%	50(28–71.8)	99.2(95.7 - 100)

NB: Values for rapid HBsAg Care and Core TM were not provided in the kit inset.

## Discussion

Hepatitis B viral infection continues to pose a major threat in many developing countries including Ghana. Few studies have attempted to describe the prevalence of HBsAg among blood donors in different parts of Ghana [4, 5]. These estimates were however based on the use of HBsAg rapid kits which could underestimate the prevalence of HBsAg infection. Using an ELISA based method (recommended gold standard); the present study showed HBsAg prevalence of 14.6% among voluntary and replacement blood donors in a rural area of Ghana. This prevalence rate is similar to a previous study done in an urban area of Ghana using an ELISA based assay [3], but higher compared to reported prevalences in some developing and developed countries [10–13].

The seeming high prevalence of HBV infection among Ghanaians could possibly suggest that not much is being done in terms of immunization and public health education of the population against the risk of transmission. This situation is likely to be prominent among individuals in rural areas where the level of poverty is very high and individuals may not be able to afford 3 shots of hepatitis B vaccination. Although the current immunization policy in Ghana has included hepatitis B vaccine as part of the childhood vaccines given to newborns, the seeming neglect of the adult population needs to be looked at in order to reduce the risk of transfusion related infections. The low prevalence rates in the developed countries are due to effective immunization and public health education [14].

In order to evaluate the hepatitis B status of blood donors, the proportion of subjects who tested positive for HBsAg were screened for other serological markers (HBeAg, HBeAb and HBcAb). Our results showed a 16.7% prevalence rate of HBeAg. This is higher compared to studies done in other African countries [15, 16] but similar to a previous study in Ghana [17].

HBeAg is one important marker that determines the replicative state of the virus and the high occurrence of this marker among HBV infected subjects could suggest that a significant proportion of the population have active HBV infections.

This could have devastating public health consequences if adequate measures are not taken to reduce HBV infections in Ghana. There is therefore the need for adequate public health education and vaccination interventions to reduce the risk of hepatitis B virus infections.

One unique finding of this study was the poor performance of all five rapid ICT kits that were evaluated. ICT kits are now widely used for quick screening of blood donors in developing countries. This method can be completed in 10–20 minutes and requires less skilled personnel for the test to be performed. It is also not expensive and hence easily used in rural and peri-urban areas for screening of blood donors. However, the influx of various brands of these kits in the Ghanaian market without subject to appropriate quality testing is worrisome and could pose risk to the health of the entire country. All five test kits evaluated in this study had sensitivities of around 50-59% with poor kappa statistics of round 60%. This poor kit performance could be due to a number of reasons such as poor storage conditions or low detection limits. We however ruled out the possibility of poor storage at the transfusion/testing centres by buying the kit brand directly from suppliers for the study. We therefore believe such poor performance of the kits could be due to low detection limits of kits designed by manufacturers or poor conditions of transport and storage of the kits by importers and retailers. Interestingly the reported sensitivities from the kit insets of some of the rapid kits varied significantly from results of our study. This could possibly suggest an inaccurate information or deception of the public by manufacturers of these kits.

Our results are not too different from other studies reported in Ghana [6, 18]. Allain et al. [6] previously reported low sensitivity of both latex agglutination and rapid dipstick compared to ELISA and PCR methods [6].

They further explained that the low sensitivity of rapid dipsticks were probably due to the low viral load of hepatitis virus among chronic carriers. In spite of this information being available almost over a decade now, many blood donation centres still rely on less sensitive rapid kits as a screening tool. One possible reason why these kits are massively patronised by Ghanaian health facilities is because of the low cost. The question we ask ourselves is should cost serve as determining factor in purchasing screening kits in Ghana? We believe it is important that appropriate kits which are sensitive and specific be used to screen blood donors so as to reduce the risk of transmission of hepatitis B virus infections.

One major limitation of the study is the small sample size of our study subjects. This was due to logistic constraints. We therefore recommend that future studies be performed on larger population size and other brands of HBsAg rapid kits be also evaluated.

In conclusion, rapid test kits or ICT kits for HBsAg may not be helpful for screening blood donor samples in Ghana due to its low sensitivity. We therefore recommend using them as first line screening kits and further confirming all negative samples using commercially available ELISA kits. The influx of rapid kits on the market should also be adequately controlled and evaluated by the standards authority to ensure that they are of high quality. These interventions if adopted could help protect the public against transfusion related infectious diseases.

## Abbreviations

HBV: Hepatitis B virus
HBsAg: Hepatitis B Surface Antigen
ELISA: Enzyme Immunosorbent Assay
EDTA: Ethylenediaminetetraacetic acid
ICT: Immunochromatographic tests
ELISA: Enzyme Linked Immunosorbent Assays.
